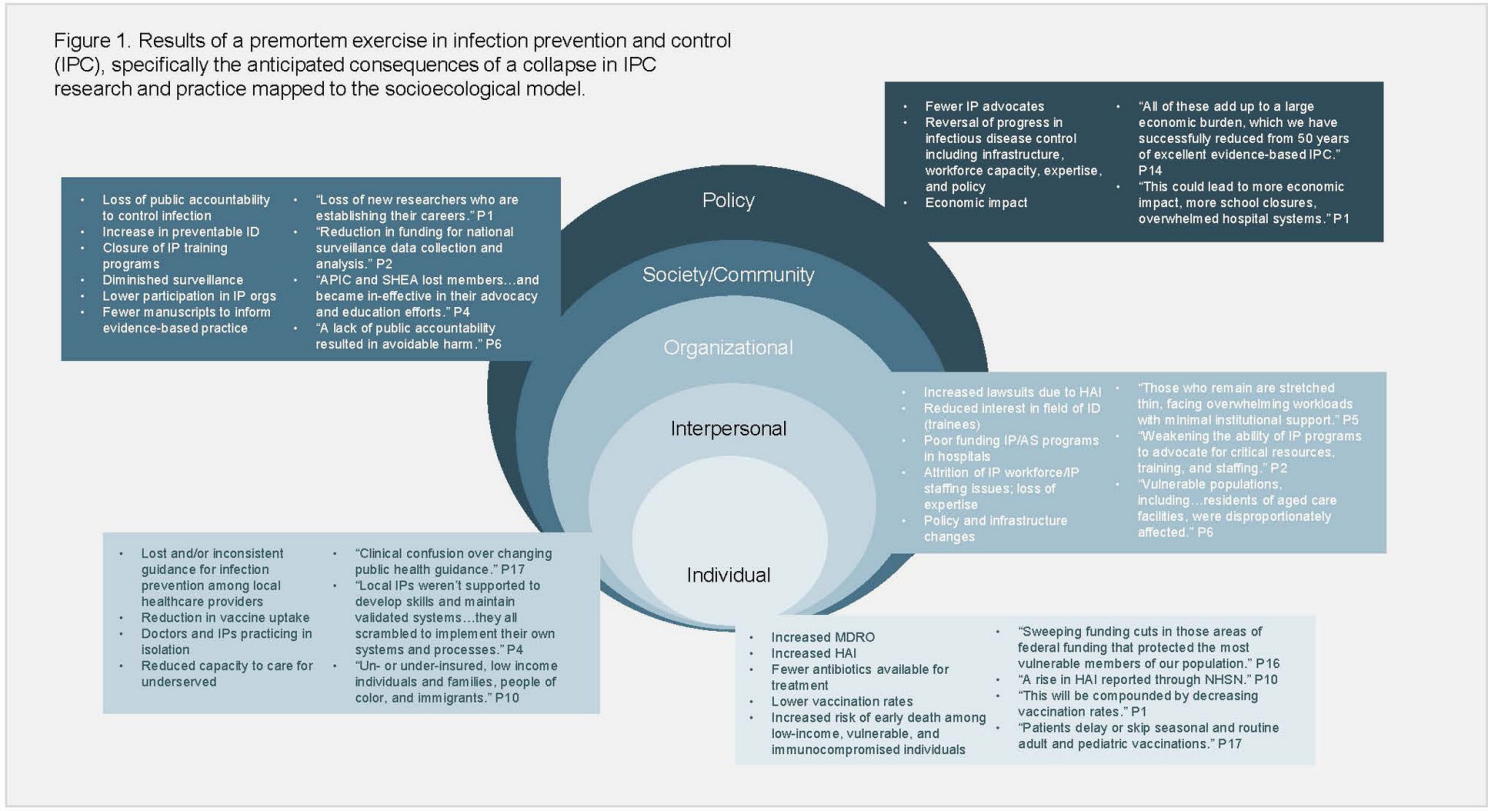# 265 National Healthcare Safety Network SSI Surveillance Misses Many Infant Congenital Cardiac Operations: How to Fix It

**DOI:** 10.1017/ash.2026.10631

**Published:** 2026-06-23

**Authors:** Stephanie Stroever, Elizabeth Monsees, Lisa Hall, Monika Pogorzelska-Maziarz, Shelley Kon, Heather Gilmartin

**Affiliations:** 1 Texas Tech University Health Sciences Center; 2 Children’s Mercy Hospital; 3 The University of Queensland; 4 Rocky Mountain Regional Veterans Affairs Hospital; 5 Denver/Seattle Center of Innovation

## Abstract

**Background:** Since January 2025, critical structural and funding changes have altered the global healthcare landscape. To respond effectively, we performed a situation premortem. Premortem methods are commonly used within the field of implementation science to identify risk factors and early warning signs for failure of complex systems. Recognizing the systems-oriented nature of participant responses, we applied the socioecological model (SEM) to organize emergent themes. Methods In April 2025, a cross-sectional electronic premortem survey was distributed to IPC thought leaders. Participants were asked to imagine the year 2029 in which IPC research and practice had collapsed and to identify contributing factors, early warning signs, barriers, populations most affected, and missed opportunities for prevention or mitigation. Responses were analyzed using thematic content analysis. Themes were organized using the socioecological model to map findings across policy, community/professional, organizational, interpersonal, and individual levels. Results Nineteen of twenty-nine people agreed to participate (66.83% response rate) from the U.S., Australia, and Singapore. The majority (n = 13) were researchers with representation from clinical practice (n = 3), policy and regulation (n = 1), or in public health or professional capacity building (n = 2). Participants anticipated widespread consequences across all SEM levels (Figure 1). At the policy level, they described diminished IPC advocacy, global reversal of progress in infectious disease control, and increased economic burden. At the community and professional level, anticipated impacts included loss of public accountability, closure of training programs, reduced surveillance capacity, and fewer scholarly outputs to inform practice. Organizational consequences included inadequate funding of infection prevention/antimicrobial stewardship programs in healthcare facilities, workforce attrition, and increased legal risk. Interpersonally, participants anticipated inconsistent guidance and confusion, reduced support for frontline clinicians, decreased vaccine uptake, and diminished capacity to serve underserved populations. At the individual level, consequences included increased multidrug-resistant and healthcare-associated infections, fewer treatment options, lower vaccination rates, and increased morbidity and mortality among vulnerable, low-income, and immunocompromised individuals. Conclusion This premortem exercise highlights the potential impacts of a collapse of IPC research and practice. Participants anticipated cascading harms across multiple levels. These results identify areas for proactive, creative strategies tailored to specific socioecological domains of impact. Reliance on traditional federal funding models and the expectation for national data collection and reporting standards may be insufficient. The field must collaborate and develop alternative approaches to sustain IPC research, training, and practice to prevent negative downstream consequences and uphold professional values.

**Figure:**